# Minimum-norm cortical source estimation in layered head models is robust against skull conductivity error^☆^^☆☆^

**DOI:** 10.1016/j.neuroimage.2013.04.086

**Published:** 2013-11-01

**Authors:** Matti Stenroos, Olaf Hauk

**Affiliations:** a MRC Cognition and Brain Sciences Unit, 15 Chaucer Road, Cambridge CB2 7EF, UK; b Department of Biomedical Engineering and Computational Science, Aalto University, P.O. Box 12200, FI-00076 Aalto, Finland

**Keywords:** Electroencephalography, Magnetoencephalography, Inverse problem, Minimum-norm estimation, Skull conductivity

## Abstract

The conductivity profile of the head has a major effect on EEG signals, but unfortunately the conductivity for the most important compartment, skull, is only poorly known. In dipole modeling studies, errors in modeled skull conductivity have been considered to have a detrimental effect on EEG source estimation. However, as dipole models are very restrictive, those results cannot be generalized to other source estimation methods. In this work, we studied the sensitivity of EEG and combined MEG + EEG source estimation to errors in skull conductivity using a distributed source model and minimum-norm (MN) estimation.

We used a MEG/EEG modeling set-up that reflected state-of-the-art practices of experimental research. Cortical surfaces were segmented and realistically-shaped three-layer anatomical head models were constructed, and forward models were built with Galerkin boundary element method while varying the skull conductivity. Lead-field topographies and MN spatial filter vectors were compared across conductivities, and the localization and spatial spread of the MN estimators were assessed using intuitive resolution metrics.

The results showed that the MN estimator is robust against errors in skull conductivity: the conductivity had a moderate effect on amplitudes of lead fields and spatial filter vectors, but the effect on corresponding morphologies was small. The localization performance of the EEG or combined MEG + EEG MN estimator was only minimally affected by the conductivity error, while the spread of the estimate varied slightly. Thus, the uncertainty with respect to skull conductivity should not prevent researchers from applying minimum norm estimation to EEG or combined MEG + EEG data. Comparing our results to those obtained earlier with dipole models shows that general judgment on the performance of an imaging modality should not be based on analysis with one source estimation method only.

## Highlights

• We tested EEG and MEG + EEG minimum-norm estimation with errors in skull models.
• Error in skull conductivity has only a small effect on morphology.
• Error in skull conductivity has moderate effect on amplitudes.
• Quality of minimum-norm estimates is minimally affected by skull conductivity error.
• Sensitivity to conductivity error depends on chosen source estimation method.

## Introduction

Electrical activity of the brain can be studied non-invasively using electro- or magnetoencephalography (EEG, MEG). EEG measures scalp potential differences of the electric field driven by the neural currents, and MEG measures the magnetic field outside the head, generated by both the neural currents and ohmic volume currents driven by the electric field (Baillet et al., 2001; Hämäläinen et al., 1993). The majority of the currents stay inside the poorly-conducting skull, and only a fraction of the electric field generated by the neural activity passes through the skull to the scalp and electrodes. The EEG signal is thus strongly influenced by the shape and conductivity of the skull (and scalp), while the MEG depends less on these features. To estimate the sources of the measured signals, the conductivity profile of the head needs to be modeled. The value of skull conductivity is, however, not well known. The sensitivity of EEG to volume conduction and the poorly-known skull conductivity are commonly considered detrimental for EEG and combined MEG + EEG (EMEG) source estimation. However, earlier studies on the topic have been restricted to dipole models only. In this work, we assess for the first time the sensitivity of EEG and EMEG source estimation to errors in skull conductivity using a distributed source model and minimum-norm estimation.

Any source estimation procedure makes use of a volume conductor model (VCM) that characterizes the relationship between the source activity and resulting signals; it thus plays a crucial role in the estimation of the neural sources of measured EEG/MEG signals. In an experimental EEG or EMEG source analysis study, the VCM typically comprises three homogeneous regions: the brain, skull, and scalp. There is considerable variance in the estimated skull conductivity: values for the ratio *K* between conductivities of soft tissues and skull range between 15 and 40 (Akhtari et al., 2002; Dannhauer et al., 2011; Lai et al., 2005; Oostendorp et al., 2000; Zhang et al., 2006). One reason for the large variation is that the concept of skull conductivity is only a practical approximation: the skull has a fine-structure of spongy and compact regions that have different conductivities (Akhtari et al., 2002; Dannhauer et al., 2011), but modeling the fine-structure is laborious and has so far not been used in experimental brain research. The effect of conductivity errors on modeled signals in sensor space has been characterized in (e.g., Haueisen et al., 1997; Vallaghe and Clerc, 2009). In Haueisen et al. (1997) it was found that differences in tissue conductivities had large effects on signal strength, while signal topographies changed less. In experimental work, also the inaccurate segmentation of the skull leads to errors in modeled signals. Effects of geometry errors on lead fields have been studied in, for example (Lanfer et al., 2012): local errors in the upper parts of the skull had a small or moderate effect on sources near the erroneous regions, and large-scale errors in the basal skull regions had a moderate or large effect on inferior sources, especially those in the cerebellum.

The sensitivity of EEG source estimation to errors in skull conductivity has so far been studied only with dipole models (e.g., Chen et al., 2010; Crevecoeur et al., 2012; Vanrumste et al., 2000), resulting in localization errors between 5 and 8 mm (Chen et al., 2010; Crevecoeur et al., 2012) and up to 28 mm (Vanrumste et al., 2000) due to the erroneous skull conductivity. The effects of simplifications and geometry errors in the skull model on dipole localization have been studied in (e.g. Dannhauer et al., 2011; Huiskamp et al., 1999; Lanfer et al., 2012; Steinstraeter et al., 2010; Vanrumste et al., 2000). Overall, the effect of geometry errors on source localization seems to be smaller than or of the same order as the effect of conductivity errors (Huiskamp et al., 1999; Vanrumste et al., 2000). In source space, geometry errors affect mostly sources close to the erroneous regions (Lanfer et al., 2012; Vanrumste et al., 2000), while conductivity errors seem to have a more global effect. Furthermore, the effects of geometry errors also depend on conductivity.

In our experience, results of existing studies seem to have been understood as general statements about the sensitivity of EEG to errors in skull conductivity or geometry. These results cannot, however, be generalized beyond dipole modeling, as there are fundamental differences across source estimation methods: in classic dipole fitting, there is a strong assumption on the nature of neural activity (single focal source) but no prior anatomical information on the location and orientation of the source, and the fitting is done iteratively with non-linear optimization. In contrast, minimum-norm estimation (Dale and Sereno, 1993; Fuchs et al., 1999; Hämäläinen and Ilmoniemi, 1984; Lin et al., 2006a,b) poses no restriction to the number or size of active brain regions, typically uses anatomical prior information on possible source locations and orientations, and is computed as a single matrix–vector operation.

In this work, we present systematic simulations to assess the sensitivity of EEG and EMEG to error in skull conductivity, when the source estimation problem is solved with linear minimum-norm estimation in a three-layer head model. To focus on the poorly-known value of conductivity, we omit other modeling errors (e.g., model simplifications, geometrical errors due to poor segmentation, and sensor coregistration errors) from this study. First, we carry out an EEG forward-model sensitivity study using realistic source anatomy. Then, for the first time, we study the sensitivity of the minimum-norm estimator to skull conductivity errors with both traditional sensitivity measures (for EEG only) and using intuitive resolution metrics (for both EEG and EMEG).

## Models and methods

The analysis was carried out using an existing data set of 14 subjects that was collected at the MRC Cognition and Brain Sciences Unit using an Elekta Neuromag Vectorview MEG/EEG scanner (www.elekta.com) in an experimental brain research project and pre-processed according to the standard analysis workflow (Hämäläinen, 2009). Of the data set, only anatomical information and noise statistics were used; the actual analysis was carried out with computer models.

### Anatomical modeling

T1-weighted anatomical magnetic resonance images were acquired with a 3T Siemens Tim Trio scanner using an MP RAGE sequence. The imaging data sets were pre-processed and the cortical surface was segmented using FreeSurfer software (Dale et al., 1999; Fischl, 2012; Fischl et al., 1999). The source space was then defined by decimating the cortical segmentation with the MNE software (Hämäläinen, 2009), resulting in source meshes for the left and right hemispheres, comprising 4098 vertices each. The boundary surfaces of the skull and scalp were automatically segmented with MNE/FreeSurfer using the watershed algorithm (Segonne et al., 2004), yielding three meshes with 2562 vertices per mesh. The 70-electrode EEG cap was prepared and the electrode locations were digitized with an electromagnetic Polhemus Fasttrak localizer (www.polhemus.com) along with the locations of preauricular points and nasion as well as about 50–100 additional points randomly distributed across the scalp. The electrodes and MEG coils were then co-registered to the MR set and scalp mesh using the MNE software.

For visualization and group analysis, average brain geometry and morphing-maps from individual brains to the average brain were constructed using FreeSurfer software and custom scripts written according to the principles presented in the MNE manual (Hämäläinen, 2009). The anatomical models were imported to Matlab (www.mathworks.com), where the rest of the modeling and analysis was carried out.

### Forward computation

Three-shell boundary-element transfer matrices were built for each subject using state-of-the-art linear Galerkin BEM with Isolated Source Approach (Stenroos and Sarvas, 2012). The conductivities of the brain, skull and scalp regions were assumed [1 1/*K* 1] × 0.230 S/m, where the skull conductivity contrast *K* had the value of 15, 25, or 40. The digitized electrode positions were projected to nearest locations on the scalp, and potential from model vertices was interpolated linearly to these positions using the BEM basis functions. The equivalent source of the EEG/MEG signal, primary current distribution ${\vec{J}}_{p}$ (Hämäläinen et al., 1993) was discretized into a set of dipoles placed into the vertices of the source meshes. For each subject and conductivity ratio, lead-field matrices *L* were built: the *i*^th^ column of the lead-field matrix is the signal topography produced by a unit-amplitude normally-oriented dipole placed in the *i*^th^ vertex of the source mesh. The zero-level of the potential was set to the mean of the potential of all electrodes. Using the lead-field matrix *L*, the signal *d* generated by a (discretized) source distribution *s* can thus be modeled as *d* = *Ls*.

### Source estimation

In linear estimation, an inverse mapping of the linear measurement model is sought:

$$d=\mathrm{Ls}+n\to \hat{s}=\mathrm{Gd}\text{,}$$

where *n* is the measurement noise and *G* is the source estimator. This problem is strongly under-determined: in our typical cortex model, the degree-of-freedom (DoF) of the estimate is 8196, while the DoF of the EEG and EMEG measurement is here only 69 and 375, respectively. A standard way for finding a unique solution is to use the minimum-L2-norm (MN) estimator (Dale and Sereno, 1993; Fuchs et al., 1999; Hämäläinen and Ilmoniemi, 1984; Hämäläinen and Ilmoniemi, 1994; Lin et al., 2006a,b), which minimizes the L2-norm of the source estimate while balancing between the reconstruction of the measured data and suppression of noise. If all source candidates are treated equally i.e. no source priors or weights are set, the MN estimator is of the form

$$G=L^{T}{\left(LL^{T}+\lambda^{2}C\right)}^{-1}\text{,}$$

where *T* is matrix transpose, *C* is an estimate of the noise covariance matrix, and *λ*^2^ is the regularization parameter that sets the balance between reproduction of measured data and suppression of noise. In our data set, *C* was constructed from measured pre-stimulus data, and *λ*^2^ was set as suggested in Lin et al. (2006a,b), assuming the mean power signal-to-noise ratio of 10. The rows of *G* are often called spatial filter vectors: projecting *d* on the *i*^th^ row of *G* yields an estimate of *s* at *i*^th^ vertex that is minimally influenced by other sources. Thus, the *i*^th^ row of *G* can be interpreted as a spatial filter that has pass-band at *i*^th^ vertex and stop-band at other source vertices.

### Metrics

The forward and inverse models were assessed using two types of metrics: direct comparisons between topographies (columns) of *L* and spatial filters (rows) of *G* built with different conductivities were carried out using relative error (RE) and correlation coefficient (CC) measures, and the overall performance of estimators *G* was characterized with resolution metrics. The RE and CC are formulated as

$$\mathrm{RE}=\frac{\left|d_{\mathrm{ref}}-d_{\mathrm{test}}\right|}{\left|d_{\mathrm{ref}}\right|}$$

$$\mathrm{CC}=\frac{d_{\mathrm{ref}}-{\bar{d}}_{\mathrm{ref}}}{\left|d_{\mathrm{ref}}-{\bar{d}}_{\mathrm{ref}}\right|}\cdot \frac{d_{\mathrm{test}}-{\bar{d}}_{\mathrm{test}}}{\left|d_{\mathrm{test}}-{\bar{d}}_{\mathrm{test}}\right|}\text{,}$$

where “ref” and “test” label the reference and test solutions, respectively, and $\bar{d}$ denotes the mean of *d*. The RE is an overall error measure, sensitive to both morphology and amplitude differences, while the CC is sensitive to morphological differences only. These measures are frequently used in the comparison of forward models and in sensitivity analysis (c.f. Haueisen et al., 1997; Stenroos and Sarvas, 2012).

The results presented in terms of RE and CC may be difficult to interpret: for example, what does the CC = 0.99 between two signal topographies or spatial filter vectors mean in practice? In an experimental source imaging study, we wish to localize one or multiple source regions and perhaps characterize the time-domain interaction between different regions. In the case of linear estimators, this performance can be assessed with resolution analysis (Grave de Peralta Menendez et al., 1997; Hauk et al., 2011; Menke, 1989; Molins et al., 2008) that characterizes the peak-localization and spatial spread of the estimators: the resolution matrix is defined *R* = *GL*, and the columns and rows of *R* are point-spread (PSF) and cross-talk (CTF) functions, respectively. The *i*^th^ PSF shows how the estimate of a unit source placed at *i*^th^ vertex spreads to the source space, while the *j*^th^ CTF shows, how unit sources in all possible positions would contribute or leak to the source estimate at vertex *j*; for an example PSF/CTF pattern, see (Hauk et al., 2011). In earlier EEG/MEG resolution analysis studies (Grave de Peralta Menendez et al., 1997; Hauk et al., 2011; Molins et al., 2008), the resolution matrix *R* was constructed assuming the estimator *G* built from the correct forward model *L*. Here we defined *R* = *G*_test_*L*_ref_ and varied the skull conductivity used in *G*_test_ If test and reference conductivities are the same, the resolution kernel of the MN estimator is symmetric and thus the PSF and CTF for a particular vertex are identical. Introducing the conductivity error (or any other error) to *G*_test_ or *L*_ref_ removes this symmetry, and PSF and CTF need to be studied separately.

The localization performance of the estimators was assessed by computing the peak position error (PPE) that measures the distance between the center-of-mass of the estimate and the true source. For a source at ${\vec{r}}_{\mathrm{true}}$,

$$\mathrm{PPE}=\left|\frac{\sum_{i}\left|{\hat{s}}_{i}\right|{\vec{r}}_{i}}{\sum_{i}\left|{\hat{s}}_{i}\right|}-{\vec{r}}_{\mathrm{true}}\right|\text{,}$$

where *i* runs through all indices, in which *s* has values above the relative threshold *t*,$\left|{\hat{s}}_{i}\right|\geq t|\hat{s}{|}_{\max}$. Here we used *t* = 0.5, focusing the metric to strong estimated amplitudes and omitting the low-amplitude values; the same metric and threshold has been used in Lin et al. (2006a,b). The spread of the estimate was characterized with spatial deviation (SD) and cortical area (CA) metrics:

$$\mathrm{SD}=\sqrt{\frac{\sum_{i}\left|{\hat{s}}_{i}\right|{\left({\vec{r}}_{i}-{\vec{r}}_{\mathrm{true}}\right)}^{2}}{\sum_{i}\left|{\hat{s}}_{i}\right|}}$$

$$\mathrm{CA}=\underset{i}{\sum }A_{i}\text{,}$$

where *A*_*i*_ is the relative cortical surface area associated with vertex *i* and *i*, again, runs through all indices for which *t*,$\left|{\hat{s}}_{i}\right|\geq t|\hat{s}{|}_{\max}$. For SD, we used the thresholds of 0 and 0.5 to distinguish between high- and low-amplitude values of the estimates, while CA was computed only with the threshold of 0.5.

## Computations and results

First, forward and inverse models were built for each subject. Using these models, metrics were computed separately for each subject and source vertex. Then, the distributions of metrics over all subjects were morphed to the average brain surface, and population mean and standard deviation of the metrics were visualized on an inflated brain surface. The analysis was done in two stages: 1) comparison of forward models and source estimators with RE and CC measures, and 2) resolution metrics for point-spread and cross-talk functions. In all comparisons across conductivities, the reference conductivity contrast was chosen *K* = 25, and the test contrasts were 15 and 40; the test contrasts are in the opposite ends of the currently accepted range, and the reference contrast is chosen so that comparisons to relevant conductivities both larger and smaller than the reference can be done. As the head anatomy and sensor geometries have high symmetry, we present results for the left hemisphere only.

### Forward model and source estimator

**Fig. 1:**
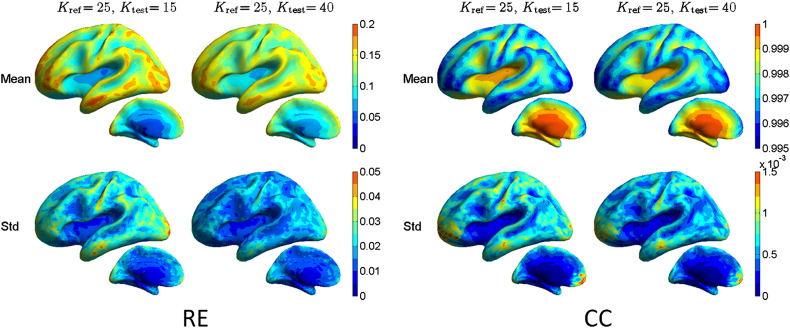
Relative error (RE) and correlation coefficient (CC) between EEG forward models computed with different skull conductivity contrasts *K*. The reference *K* is in all comparisons 25, while the test *K* is either 15 or 40. The larger and smaller plots show the lateral and medial views of the inflated brain surface, respectively.

The effect of skull conductivity on signal topographies (columns) of the forward models *L* and spatial filter vectors (rows) of source estimators *G* was assessed using relative error (RE) and correlation coefficient (CC) measures: *L* and *G* were built using different skull conductivities (*K*_ref_ = 25, *K*_test_ = 15 or 40). For each source vertex, the topographies of *L* and spatial filter vectors of *G* were compared across conductivities. The population means and standard deviations of both metrics for the forward solutions for all source positions are shown in Fig. 1. The results show that the largest range of errors is obtained, when the skull conductivity is overestimated in the inverse model (*K*_ref_ = 25, *K*_test_ = 15); the overall RE, presented as “mean ± standard deviation,” is 0.11 ± 0.04 and the CC 0.998 ± 0.0013. With underestimated conductivity (*K*_ref_ = 25, *K*_test_ = 40), the corresponding numbers are RE 0.12 ± 0.03 and CC 0.998 ± 0.0012. The morphologies of the RE plots for over- and underestimated *K* are very similar and the CC plots are almost identical. As a function of source depth, the smaller and larger errors are obtained with deep and superficial sources, respectively. The morphological errors of the topographies are in the same range with results presented in Haueisen et al. (1997).

**Fig. 2:**
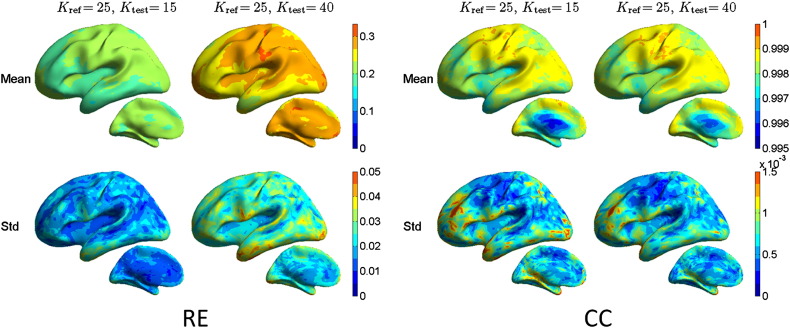
Relative error (RE) and correlation coefficient (CC) between EEG spatial filter vectors with different skull conductivity contrasts *K*. The reference *K* is in all comparisons 25, while the test *K* is either 15 or 40.

Corresponding results for the spatial filter vectors are shown in Fig. 2. The largest errors are obtained, when the conductivity is underestimated (*K*_test_ > *K*_ref_): RE has then the overall mean 0.27 ± 0.03, and the corresponding CC is 0.998 ± 0.0009. For the overestimated conductivity, the RE is 0.21 (0.02) and the CC is 0.998 ± 0.0010. The results thus show that, compared to the modeled signal topographies, the conductivity error has overall the same morphological effect and larger amplitude effect on the spatial filter vectors. In the CC plots, however, the behavior as function of source depth is different from the forward model: for source estimator, deep sources tend to have larger errors. In addition, we directly compared minimum-norm estimates of simulated point sources; also that analysis showed moderate amplitude errors and small morphological errors (RE < 0.3, CC > 0.995). The high CC between topographies or spatial filter vectors computed with different conductivities means that the errors indicated by the RE measure are mainly of amplitude, but not of morphological, nature.

### Resolution metrics

In the main part of the analysis, we studied the localization and spread properties of the MN estimator using different skull conductivities. First, resolution matrices *R* = *G*_test_*L*_ref_ were built, using *K* = *K*_f_ = 25 for the *L*_ref_ and *K* = *K*_i_ = 15, 25 or 40 for the *G*_test_. Then, metrics were computed for point-spread and cross-talk functions with all test conductivities; the metrics were thus computed with correct, overestimated, and underestimated skull conductivities. As the results for corresponding PSFs and CTFs were very similar, we present the results for the point-spread functions only; the corresponding plots for the CTFs are available in the supplementary material. Here we show the results for EEG; the corresponding plots for the EMEG are presented as an Appendix A. However, the following analysis applies to both EEG and EMEG, as the overall effects of conductivity errors were the same for both modalities. With respect to the aim of this work, the essential information lies in the relative differences or similarities between the metric distributions obtained with different test conductivities.

**Fig. 3:**
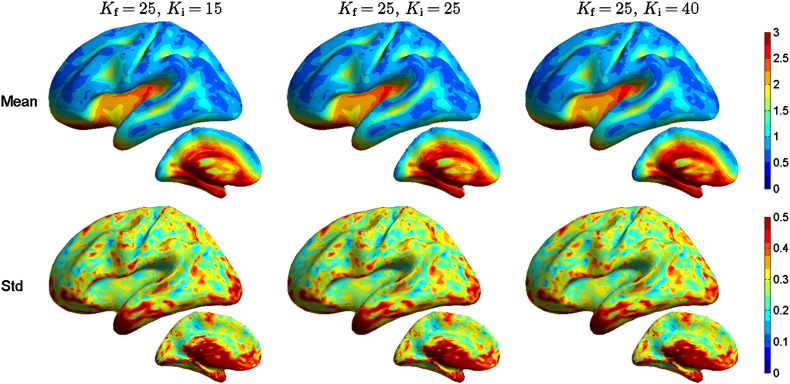
Peak position error PPE (in centimeters) for different EEG inverse model conductivities. The forward solutions were computed with *K* = *K*_f_ = 25 and the inverse solutions with *K* = *K*_i_ of 15, 25, or 40. The pseudocolor plots show the population mean on the upper row and standard deviation on the lower row with all test conductivities (columns). The results with the reference model, computed with the same conductivities in the forward and inverse models, are in the middle column.

The peak position error (PPE) is shown in Fig. 3. The population means and standard deviations obtained with different test conductivities are almost identical; the relative change of PPE due to the conductivity error is small. The overall PPE in the reference case is 15.4 ± 8.0 mm, and in the cases of over- and underestimated *K*, the overall change of PPE is − 0.09 ± 0.30 mm and 0.14 ± 0.35 mm, respectively. The largest absolute changes, of the order of ± 2.5 mm, are obtained in the cingulate cortex and subcortical areas, where the PPE of the reference model is around 25 mm. The skull conductivity error has thus negligible overall effect on the localization performance of the MN estimator.

**Fig. 4:**
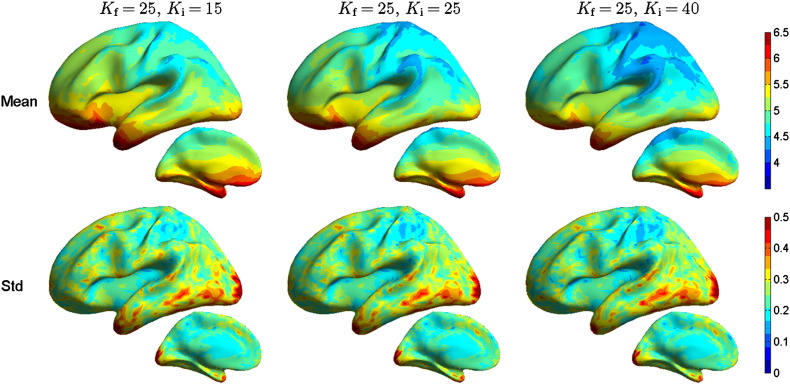
Spatial deviation (in centimeters) for EEG as function of test model conductivity. Notice that the color scale for the mean does not start at zero. For further explanation, see the caption of Fig. 3.

**Fig. 5:**
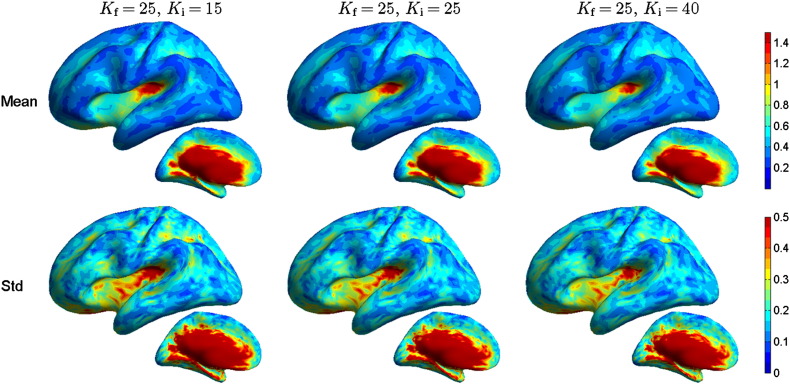
Relative cortical area CA (in percents) for EEG as function of test model conductivity. For further explanation, see the caption of Fig. 3.

The unthresholded SD results are shown in Fig. 4. The results show that choosing *K*_i_ < *K*_f_ slightly increases the spread and *K*_i_ > *K*_f_ decreases it. However, when the metric was thresholded at 50% of maximum (plots not shown), this effect was removed, and plots with all conductivities looked the same; thus the differences in the metrics of Fig. 4 are due to the low-amplitude values of the source estimates. The CA metric is displayed in Fig. 5; the plots with and without conductivity errors look again almost identical. The results thus show that the spread of high-amplitude values of the source estimates is not affected by the conductivity error, while over- and underestimating the *K* slightly increases and decreases, respectively, the low-amplitude ripple of the estimates.

## Discussion

We assessed the sensitivity of EEG and EMEG minimum-norm source estimation to modeling error in skull conductivity in a three-layer head model. The results showed clearly that the MN estimator is robust against errors in skull conductivity: the conductivity error has a moderate effect on the overall amplitude of the lead fields, spatial filter vectors and inverse solutions, but the effect on the corresponding morphologies is small. The localization performance of the MN estimator is only minimally affected by the conductivity error, while the low-amplitude spread of the estimate may vary slightly. Thus, the uncertainty with respect to skull conductivity should not prevent researchers from applying minimum norm estimation to EEG or combined MEG + EEG data.

### The role of amplitude error

Analysis of the forward models and source estimators shown in Section 3.1 showed that the skull conductivity error may cause an amplitude error of a few tens of percents. As the minimum-norm estimator renders the solution unique by giving an estimate that satisfies the data with minimal source amplitude, the total amplitude of the estimate is often much smaller than the real source amplitude: for example, for a focal source with unit amplitude, the resulting minimum-norm estimate is often widely spread, with the total amplitude of the order of 10^− 3^. The MN estimator is thus not accurately reconstructing the amplitude or extent of the source distribution. An additional overall amplitude error does thus not seem to have any practical effect.

**Fig. A1:**
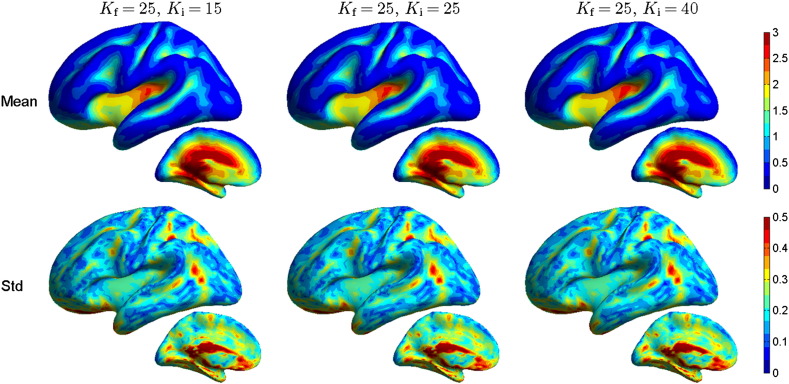
Peak position error PPE (in centimeters) for EMEG with different inverse model conductivities. For further explanation, see the caption of Fig. 3.

**Fig. A2:**
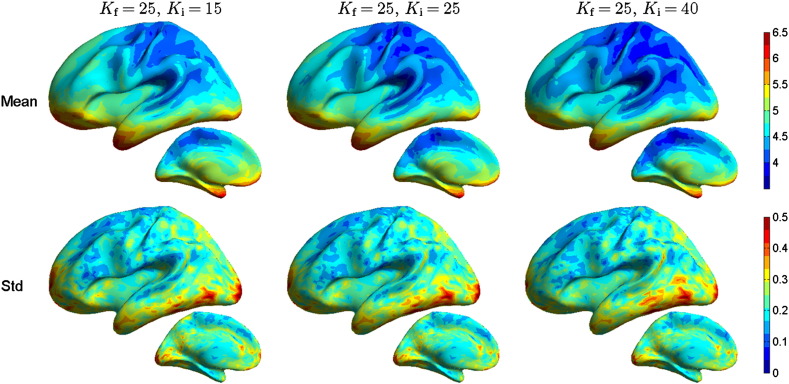
Spatial deviation (in centimeters) for EMEG as function of test model conductivity. For further explanation, see the caption of Fig. 3.

**Fig. A3:**
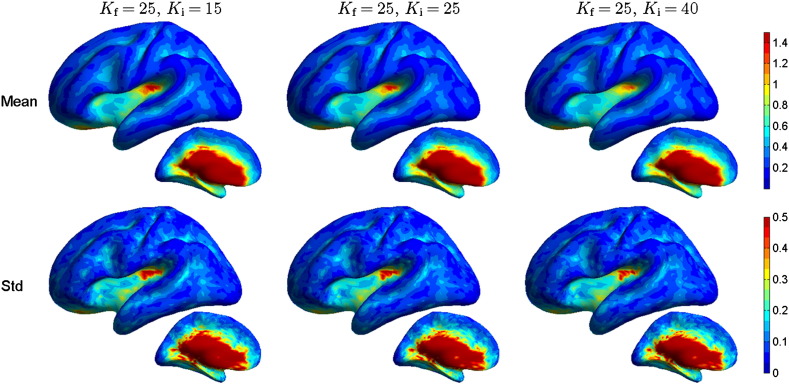
Relative cortical area CA (in percents) for EMEG as function of test model conductivity. For further explanation, see the caption of Fig. 3.

As the amplitude error depends on source position, it would be logical to think that it would corrupt source estimation, if multiple sources are simultaneously active in regions with different amplitude errors. This effect can be assessed by studying cross-talk functions that show how much sources at each position contribute to the estimate at the target position. Our cross-talk analysis gave results very similar to those presented for point-spread functions in Figs. 3, 4, 5 and A1–A3: the change of the CTFs due to the conductivity error was small (see the supplementary material for visualizations). Thus, the amplitude errors do not seem to corrupt the source estimation.

In this work, we combined EEG and MEG by using the noise covariance matrix *C* as regularization operator. This is equivalent to converting the forward model and signals to signal-to-noise ratios by pre-whitening with matrix *W = C*^− 1/2^. As the skull conductivity error causes larger amplitude errors to the lead fields of EEG than of MEG, it changes the relative scaling between EEG and MEG. According to results shown in the Appendix A, this scaling error does not seem to have affected the source estimation. Liu et al. (2002) studied the effect of a similar (constant) scaling parameter on cross-talk and reported that the scaling needs to be correct within a factor of two; the amplitude errors obtained in this study are well within that limit.

### Comparison to previous results obtained with dipole models

At first look, our results are in contrast with those obtained with dipole modeling: localization errors of up to 28 mm due to wrong skull conductivity have been presented (Vanrumste et al., 2000). Those extreme results were, however, obtained with a very large conductivity difference: data were simulated with *K*_f_ = 16 and inverted with *K*_i_ = 80. There now seems to be consensus that the contrast value of 80 is too large (c.f. Oostendorp et al., 2000) — more recent dipole studies that used smaller conductivity differences indeed reported errors of only 5 to 8 mm (Chen et al., 2010; Crevecoeur et al., 2012). Our presented results were computed with *K*_f_ = 25 and *K*_i_ = 15 or 40. We also computed metrics with *K*_f_ = 40 and *K*_i_ = 15 or 80: the population means were nearly identical to those presented in Section 3.2 and Appendix A, while the variation among subjects was slightly larger. Thus, the MN estimator retains its robustness also in the presence of untypically large conductivity errors.

A dipole-fitting algorithm searches the source-position with best-matching signal topography, testing one position at the time, while the linear minimum-norm estimator projects the weighted signal on topographies of all source candidates, minimizing the source amplitude. Due to its distributed nature and pre-fixed source positions, the MN estimator is thus less sensitive to local topography errors. On the other hand, even in ideal modeling conditions, the MN estimator produces smeared estimates and also suffers from depth bias (Lin et al., 2006b). Dipole fitting and minimum-norm current density estimation are two very different approaches for tackling with the ill-posedness of the EEG/MEG source estimation problem: in dipole-fitting, strong prior assumptions are made, leading to potentially high accuracy but lack of robustness, while in MN estimation some of the accuracy is traded for generality and robustness.

Our results show that earlier results on the role of skull conductivity obtained with dipole modeling cannot be generalized to distributed source models. It is, however, essential to stress that also our results are not general statements: the effect of model errors and inaccuracies on source estimation depends on the chosen source model and estimation technique. General assessment about the performance of an imaging modality should thus not be based on analysis with one source estimation method only. Instead, making comparable sensitivity studies across different source estimation methods would yield important information on method robustness.

### Model details

The type and level of detail of the anatomical model were chosen according to state-of-art practices in experimental brain research, guaranteeing the relevance of the results for an experimental neuroscientist. In the standard minimum-norm estimation palette, there are some choices to be made with respect to the forward solver, the segmentation of the skull and the constraining of source orientation: our analysis was carried out with the Galerkin BEM, but the results presented apply also to other numerical methods like finite-difference or finite-element methods (FDM, FEM). For segmentation, we used the default pipeline of MNE and FreeSurfer, in which the inner and outer skull surfaces are based on the envelope of the brain and shape of the scalp, respectively, leading to rather smooth skull surfaces with unrealistic shape of the lower parts of the skull. To verify the possible effect of this artifact, we also analyzed one case using a more accurate skull model based on multi-angle FLASH MR set that has better skull contrast; the effect of the conductivity error was essentially the same. For source analysis, we chose to use constrained source orientation. In experimental work, a loosely-constrained or free orientation (Lin et al., 2006a) is, however, often preferred, as it is considered more robust against errors in cortex segmentation. We carried out all resolution analysis also with free source orientation — the effect of conductivity on estimator performance was even slightly smaller than in the case of constrained source orientation.

### Future directions

Our results showed that the value of skull conductivity does not have a significant role in minimum-norm source estimation if a realistically-shaped three-shell model is used. Thus, for users of MN estimation, it does not seem necessary to, for example, try to estimate individual skull conductivity as suggested in (Chen et al., 2010) or to develop correction techniques like that reported in (Crevecoeur et al., 2012). Instead, the next logical step towards understanding the effects of volume conductor model errors on source estimation would be to study the effects of geometrical errors in the skull model and extend the analysis beyond the three-layer model by including the skull fine-structure, for both EEG and MEG. Some studies in that direction for EEG forward problem and dipole-modeling have already been done, (c.f., Huiskamp et al., 1999; Dannhauer et al., 2011; Lanfer et al., 2012), but more work is needed in order to find out, whether the cost and effort of more detailed modeling is warranted in brain research.
