# Supplementary material for: Minimum-norm cortical source estimation in layered head models is robust against skull conductivity error
Source: Neuroimage. 2013 Nov 1;81:265–72. doi: 10.1016/j.neuroimage.2013.04.086 (PMC3915841; doi:10.1016/j.neuroimage.2013.04.086)
Supplement: Supplementary file 1 — Supplementary Figures [file mmc1.docx]

# Supplementary material: resolution metrics for cross-talk functions

## EEG


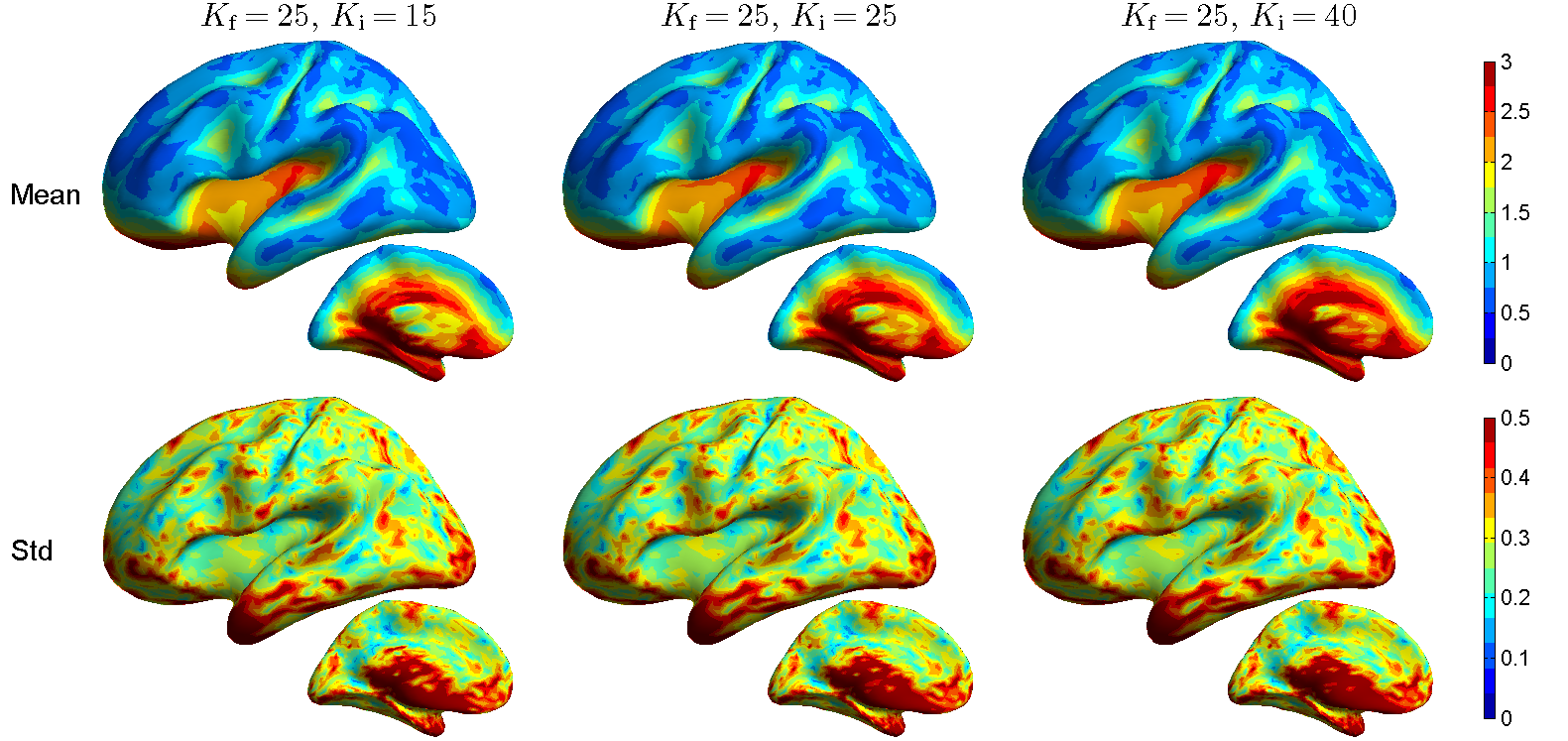


Figure S1: Peak position error PPE (in centimeters) for EEG cross-talk functions with different test model conductivities. The forward solutions were computed with *K* = *K*_f_ = 25 and the inverse solutions with *K* = *K*_i_ of 15, 25, or 40. The pseudocolor plots show the population mean on the upper row and standard deviation on the lower row with all test conductivities (columns). The results with the reference model, computed with the same conductivities in the forward and inverse models, are in the middle column.


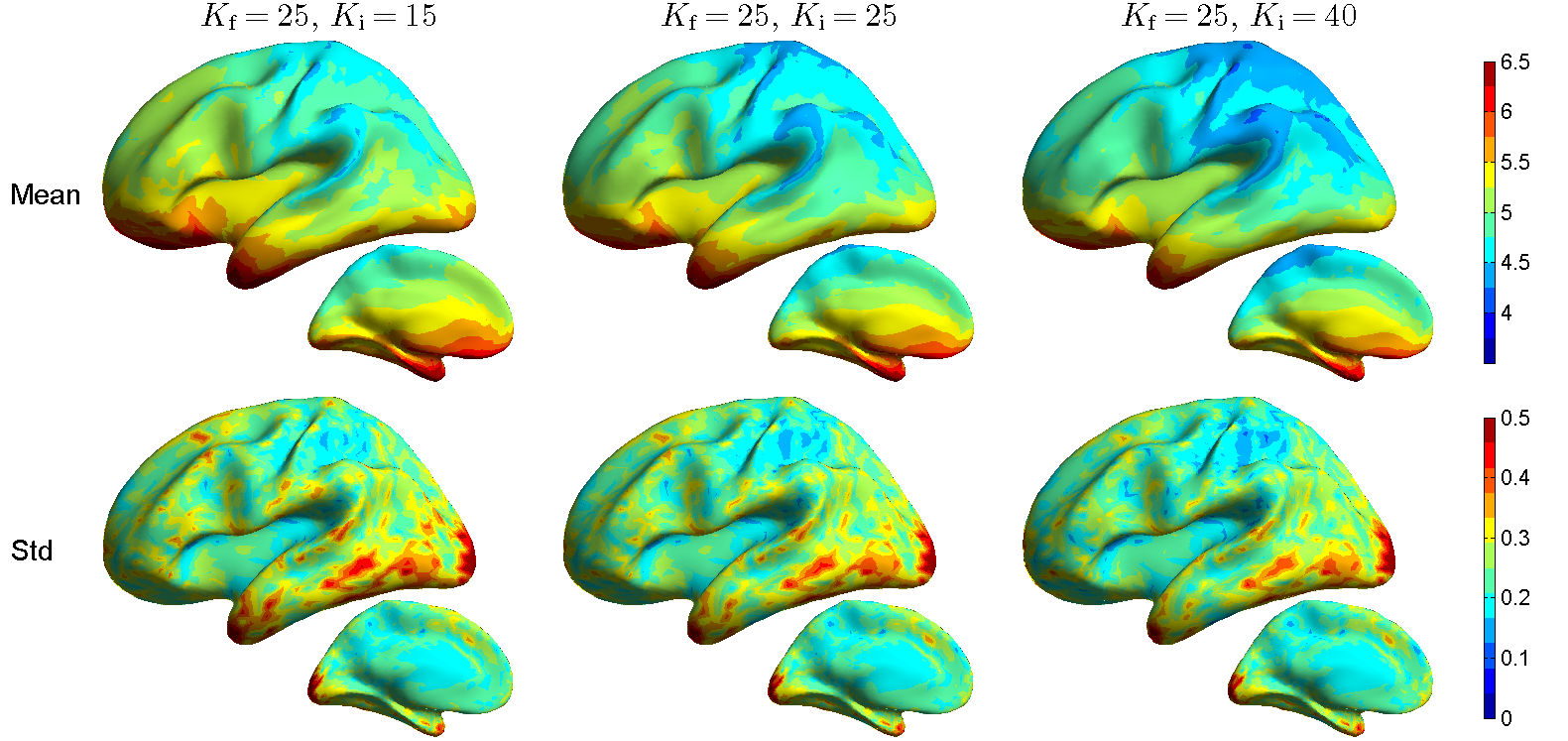


Figure S2: Spatial deviation (in centimeters) for EEG cross-talk functions with different test model conductivities. Notice that the color scale for the mean does not start at zero. For further explanation, see the caption of Fig S1.


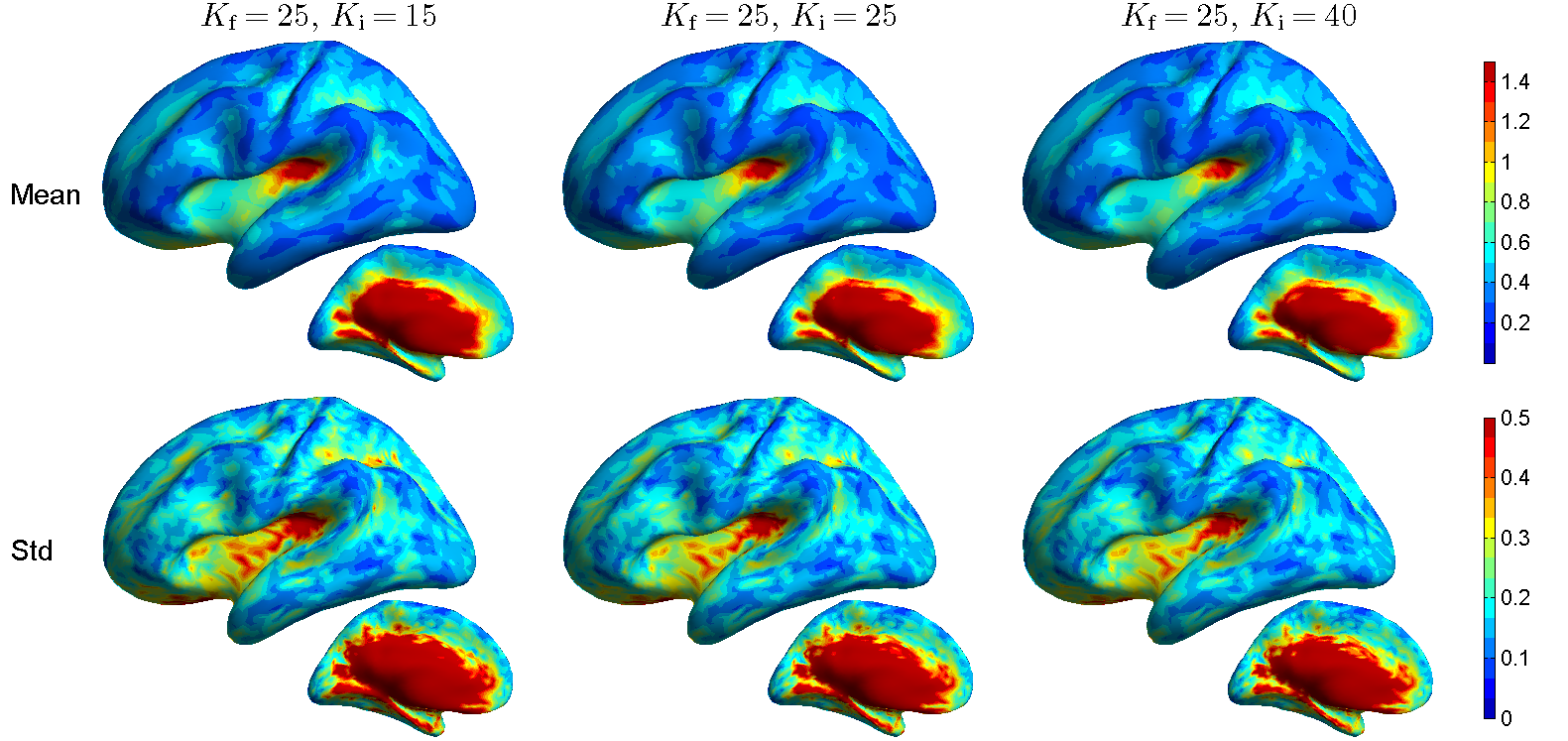


Figure S3: Relative cortical area CA (in percents) for EEG cross-talk functions with different test model conductivities. For further explanation, see the caption of Fig S1.

## Combined MEG+EEG


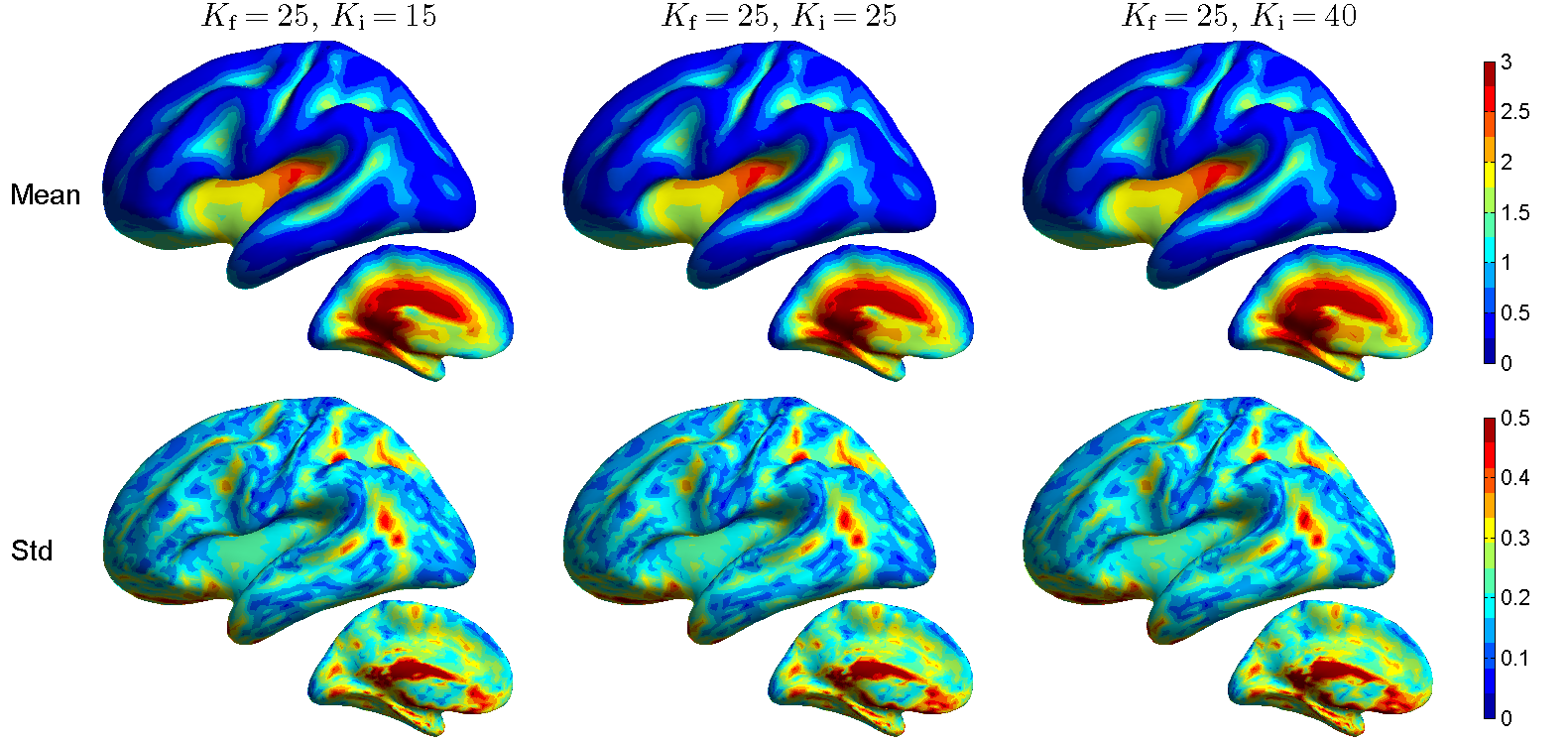


Figure S4: Peak position error PPE (in centimeters) for EMEG cross-talk functions with different test model conductivities. For further explanation, see the caption of Fig S1.


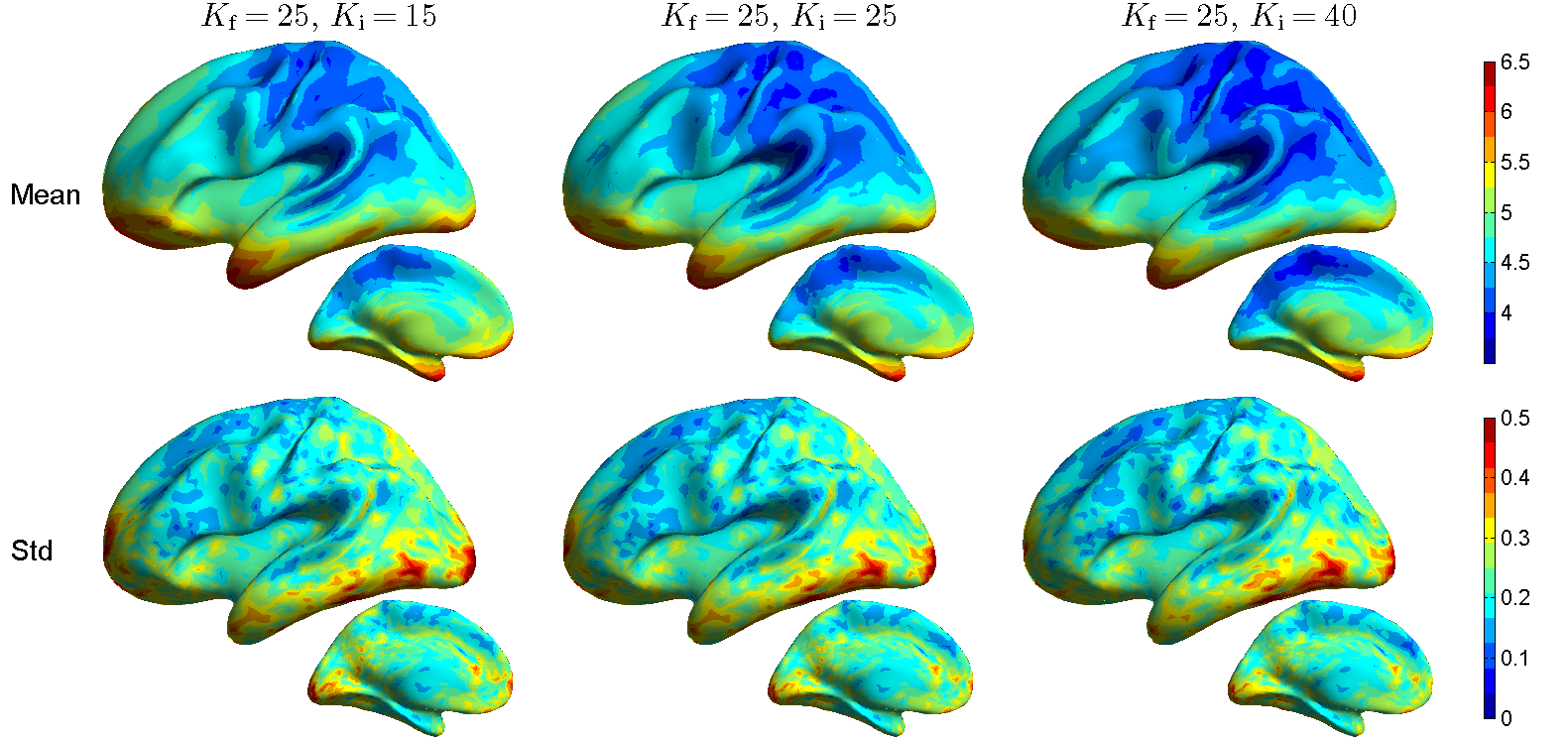


Figure S5: Spatial deviation (in centimeters) for EMEG cross-talk functions with different test model conductivities. Notice that the color scale for the mean does not start at zero. For further explanation, see the caption of Fig S1.


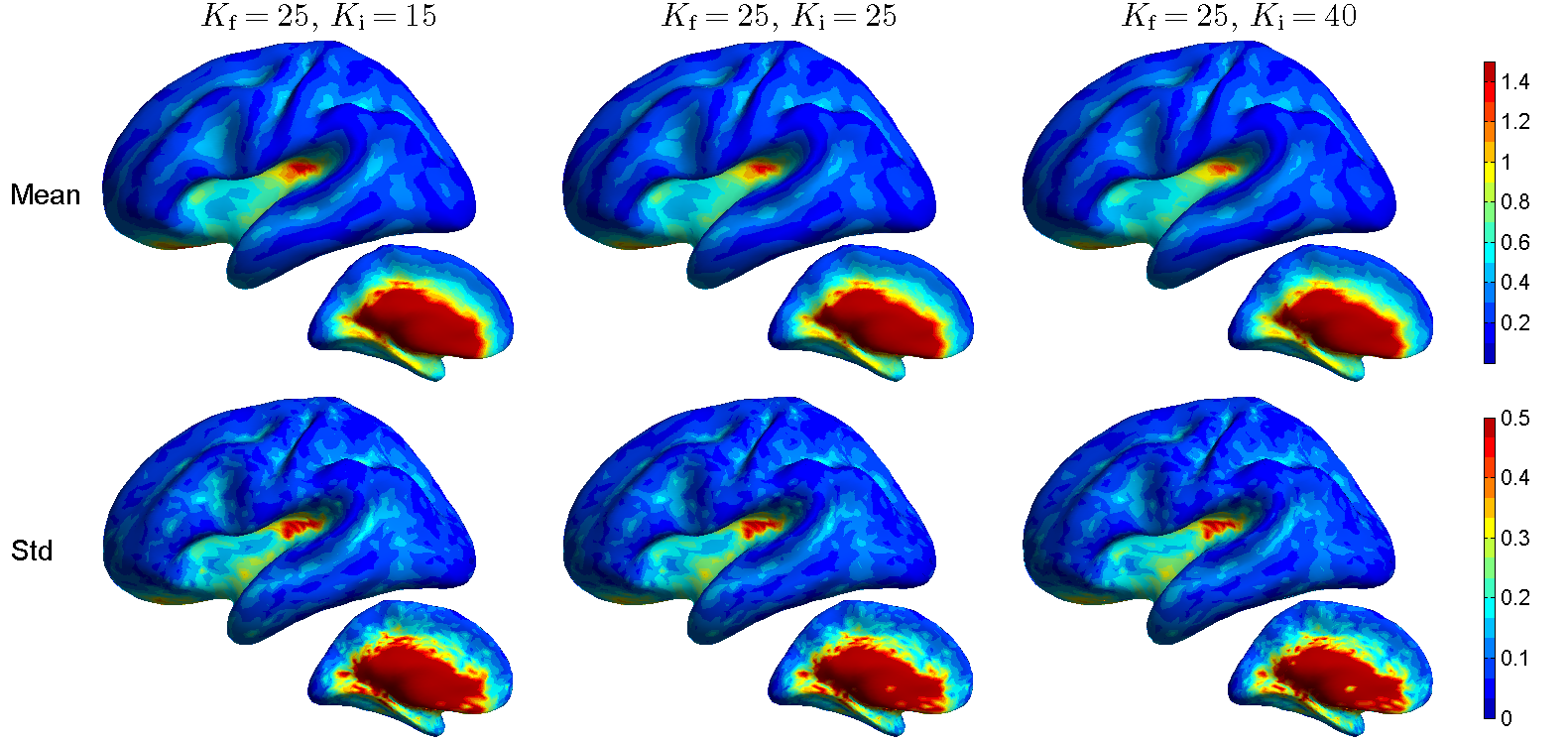


Figure S6: Relative cortical area CA (in percents) for EMEG with different test model conductivities. For further explanation, see the caption of Fig S1.
